# Community work strategies and support needs of social workers in providing substance use services to young people

**DOI:** 10.4102/hsag.v30i0.3022

**Published:** 2025-07-09

**Authors:** Jan Masombuka, Faith Mathibela

**Affiliations:** 1 Department of Social Work, Faculty of Human Science, University of South Africa, Pretoria, South Africa

**Keywords:** community work, strategies, social workers, support needs, substance use, young people

## Abstract

**Background:**

Substance use among young people is a pressing concern both globally and in South Africa. Thus, social workers, as frontline workers, employ community work strategies in assisting young people grappling with substance use. Equally, they need support in their efforts to provide effective substance use services to young people.

**Aim:**

The study aimed to explore community work strategies of social workers in providing substance use services to young people. In addition, the study explored the needs of social workers providing substance use services to young people.

**Setting:**

The study was conducted at 10 service points of the Department of Social Development (DSD) within the City of Tshwane Metropolitan Municipality (CTMM).

**Methods:**

A qualitative research approach was employed, characterised by an exploratory and descriptive design. A purposive sampling method was implemented to recruit a sample of 11 social workers from the 10 service points operated by the DSD in Gauteng, located within the CTMM. Subsequently, individual face-to-face semi-structured interviews with an interview guide were used to collect data. Thus, Tesch’s framework for qualitative data analysis was utilised.

**Results:**

Two themes emerged: Community work strategies of social workers and support needs of social workers.

**Conclusion:**

Awareness campaigns, collaboration with local stakeholders and community dialogues are the backbone of social workers’ community work strategies. Additionally, social workers need support to provide substance use services to young people effectively.

**Contribution:**

This study seeks to address a gap in understanding the community work strategies and support needs of social workers who provide substance use services to young people.

## Introduction

The prevalence of substance use among young people is a significant global concern, and South Africa is no exception to this troubling trend (Shadung, Mbedzi & Skhosana 2024). Social workers, as integral members of a multidisciplinary team, frequently serve as the initial point of contact for young people undergoing treatment for substance use (Khosa & Ndou 2022). Thus, this reality raises important questions regarding the community work strategies employed by social workers, as frontline workers, in providing substance use services to young people. Equally, it is also important to tap into the support needs of these social workers, who serve an important function in providing substance use services to young people.

Globally, young people are identified as high risk for substance use (World Drug Report 2024a). Accordingly, in Australia, 60% of people in treatment for substance use are young people under the age of 35 (World Drug Report 2024a). In addition, Africa is witnessing a significant rise in young people’s involvement with various substances, as noted in the African Union Plan of Action on Drug Control and Crime Prevention (2019–2023). Consequently, cannabis and synthetic drugs have emerged as critical challenges that necessitate collective efforts and proactive responses across the continent. Accordingly, Africa has a concerning issue with substance use, as it holds the highest percentage of young people currently receiving treatment for substance use (World Drug Report 2024b). This troubling trend underscores the urgent need for effective intervention and support systems to address the substance use challenges faced by young people on the continent.

According to the National Drug Master Plan, 2019–2024, young people in South Africa are the most vulnerable population, as they are hit hard because of the alarming rate of substance use (Department of Social Development [DSD] 2020). In February 2025, the South African Community Epidemiology Network on Drug Use (SACENDU) published a report that revealed alarmingly high rates of treatment admissions attributed to cannabis use among young people aged 18 and younger across the nation (SACENDU 2025). These data are an example of a growing crisis surrounding substance use among young people, shedding light on the rising challenges that young people in South Africa face regarding substance-related issues. The findings call for social workers to intensify their efforts to address these pressing concerns. According to the study by Carelse and Green (2019), South Africa’s high prevalence of substance use ranks it among the top 10 countries globally for substance use. As a result, the demand for social work services aimed at substance use intervention has surged in recent years. This increase prompts significant questions regarding the implications for the support services available for young people.

A related study exploring community-based initiatives designed to prevent and combat drug abuse in a South African township (Machethe, Obioha & Mofokeng 2022) emphasised the significant impact of various community strategies. It highlighted community outreach programmes that actively engage residents, youth support initiatives that provide guidance and resources to young people within community-based organisations and comprehensive drug awareness campaigns that aim to educate community members. These approaches have effectively addressed and reduced substance use within South African townships, fostering a healthier and more supportive community environment. Mathibela (2024) highlights the importance of social workers initiating and maintaining continuous preventative awareness campaigns. These campaigns are essential for equipping parents with the knowledge and understanding necessary to support young people effectively. By educating parents about their vital roles and functions, social workers can foster a more nurturing and responsive environment that promotes the well-being of young people.

Khanyi and Malesa (2022) highlight the opportunity to improve the existing substance use interventions to enhance the quality of treatment provided to young people. Thus, the study recommends providing tailored support to social workers, empowering them in their professional roles. Additionally, the study by Shadung et al. (2024) highlights the need for more social workers and supervisors in substance use. This increase in personnel is essential for ensuring adequate manpower and proper supervision. Moreover, involving the parents of young people who receive social work services is important.

The study by Madisha (2019) highlighted the need for social workers to receive support in the form of specialised training to implement substance use services for young people effectively. By enhancing their competencies, social workers can better address the unique challenges faced by young people with substance use issues. Accordingly, Sekgobela (2021) urged social service organisations to support social workers with adequate supervision in line with the supervision framework. This support will help ensure that social workers receive the guidance and resources they need to excel in their roles and effectively assist young people using substances.

Ecosystems theory was utilised to gain insights into the community work strategies and support needs of social workers providing substance use services to young people. This theory posits that individuals require effective interactions with their environment to survive and thrive (Kirst-Ashman 2017). The social environment encompasses individuals, groups, organisations and systems in which a person engages. Ecological systems theory highlights that individuals are not isolated; they are interconnected with various environmental systems, including the microsystem, mesosystem, exosystem, macrosystem and chronosystem (Ettekal & Mahoney 2017). The adoption of ecosystems theory is regarded as an appropriate framework to assist social workers in providing substance use services to young people. This framework fosters a responsive environment that enables clients to receive support and empowerment, enhancing their social functioning and promoting overall well-being.

Given this background, the study aimed to explore community work strategies of social workers providing substance use services to young people. In addition, the study explored the support needs of social workers providing substance use services to young people. By exploring these facets, the study seeks to uncover key insights that can inform the development of effective community work strategies and support systems for social workers, ultimately enhancing their ability to assist young people in addressing and overcoming substance use issues.

## Research methods and design

### Study designs and setting

A qualitative research approach was utilised to ensure an alignment with the purpose of the study. Thus, the qualitative research approach explores how individuals perceive and construct their understanding of the world based on their lived experiences (Rezaul 2019). To achieve this, explorative and descriptive research designs were adopted, enabling the exploration of the community work strategies and support needs of social workers providing services to young people. By doing so, the study shed light on the community work strategies and support needs of social workers providing services to young people. Essentially, the study utilised the 10 service points of the Gauteng DSD within the City of Tshwane Metropolitan Municipality (CTMM) as the setting. This setting provides a unique context for understanding the phenomenon under investigation. Accordingly, the CTMM is confronted with the issue of substance use, which has a detrimental effect on social cohesion and the development of stable communities (Motabogi 2022).

### Population and sampling

The study comprised social workers in the Gauteng DSD within the CTMM. Thus, population refers to the number of individuals with similar qualities and characteristics identified for a specific research study. This group encompasses those eligible for inclusion in the study (Pandey & Pandey 2015). Given the qualitative nature of the study and the limitations of time and finances, it will not be feasible to include the entire population in the study. As a result, a sample, which refers to a group of individuals selected from a larger population for measurement, was considered (Babbie 2017; Schmidt & Brown 2015). A purposive sampling method was employed to recruit 11 social workers from the 10 service points of the DSD in Gauteng within the CTMM. Accordingly, the assistance of the social work manager, as a gatekeeper in the 10 service points, was sought in recruiting participants. Subsequently, the social work manager identified two supervisors in the 10 service points within CTMM as mediators to assist with participant engagement and the recruitment of social workers. Thereafter, face-to-face meetings were scheduled with the respective social workers who gave permission to be contacted and who were officially introduced and presented with the purpose of the study. To ensure that only relevant data were collected, the researchers relied on the mediators’ information, which was later verified through biological questions during data collection.

Importantly, the sample size was not predetermined; instead, the study was guided by the principle of data saturation to ensure comprehensive data collection. Thus, the sample inclusion criteria entailed only social workers employed at the 10 service points of the DSD in Gauteng, within the CTMM, who had provided services for more than two years and were registered with the South African Council for Social Service Profession.

### Data collection

Data were gathered through individual, face-to-face, semi-structured interviews, employing an interview guide to facilitate a thorough and consistent data collection. Thus, the structure of the interview questions was based on the researchers’ experience and understanding of the topic. Accordingly, semi-structured interviews are generally conducted face-to-face, allowing the researcher to obtain valuable insights, ask relevant questions and encourage participants to share more information (Moser & Korstjens 2018). All interviews were conducted by the researchers at the social worker’s office and were digitally recorded with the consent of the participants. The interviews were conducted in English; each session lasted approximately 40 min–60 min, and field notes were taken. Essentially, the researchers are qualified social workers with respective postgraduate qualifications and are experts in qualitative research. Accordingly, the researchers were independent of the study participants, ensuring their impartial and unbiased interactions. The data collection was finalised after interviews with 11 participants, at which point data saturation was attained. It is worth noting that the interviews were conducted between November 2018 and January 2019.

### Data analysis

In accordance with the qualitative nature of the study, Tesch’s framework for qualitative data analysis was utilised (Creswell & Creswell 2018). The researchers transcribed the audio-recorded semi-structured interviews immediately after the interviews, to enable a thorough analysis of the participants’ responses. Following the completion of the transcription process, the researchers sought the expertise of an independent coder to assist in the data analysis. Thereafter, researchers consulted with the independent coder to clarify the identified themes. Consequently, the data were meticulously analysed, and the services of an independent coder were sought. Subsequently, consensus discussions were conducted to establish the themes. Following this extensive and detailed process, eight significant themes have emerged, each representing key insights derived from the research findings.

### Measures of trustworthiness

Accordingly, specific strategies were employed to ensure internal validity, as stipulated by Creswell and Creswell (2018). Data triangulation was achieved by collecting data from multiple sources, including different social workers employed by the DSD at 10 service points within Gauteng Province. The principle of peer examination was observed by ensuring that experts in the field of qualitative research were consulted throughout the study. In addition, the researchers were conscious of their biases and journaled them when necessary. Significantly, the services of an independent coder were sought to authenticate the themes presented as research findings.

### Ethical considerations

The necessary permissions to conduct the study were formally requested and obtained from the North-West University Research Ethics Regulatory Committee, which ensures that all research adheres to ethical standards. Approval was also sought and granted by the provincial office of the DSD, which oversees the region’s social welfare initiatives and programmes. This dual approval process underscores the commitment to conducting the research responsibly and ethically soundly (Ethics no: NWU-00025-18-A1). Equally, the ethical principle of informed consent was observed by ensuring that written consent was obtained from individual participants before data collection commenced. In addition, anonymity, privacy and confidentiality were observed by ensuring that pseudonyms instead of the participants’ real names were used to write up the research findings, so the data collected could not be linked to any specific participant.

## Research results

The following discusses the findings derived from data analysis involving 11 social workers. An overview of the biographical data will precede the presentation and analysis of the themes identified during the interviews with the research participants.

### Biographical data of the participants

Every participant in the study successfully attained a Bachelor of Social Work degree, a significant achievement that underscores their rigorous education and training. This degree signifies that they have received adequate training in social work and, thus, understand the principles and practices crucial for effective social work. In South Africa, this qualification is aligned with global social work education and training standards, enabling registration to practice as a social worker (Council on Higher Education 2015). Concerning the race, it is noteworthy that all participants were of black African descent. Accordingly, the black African population is the majority (51 million), constituting approximately 82% of the total South African population (Statistics South Africa 2024). Regarding the gender of participants, 10 out of 11 participants were females, while one participant was a male. Accordingly, more females than males are involved in social work in the treatment centres in the City of Tshwane, Gauteng (Mathibela 2024).

## Discussion of themes

The thematic analysis identified two primary themes, each accompanied by several subthemes. The first theme outlines the community work strategies of social workers providing substance use services to young people. The second theme addresses the support needs of social workers in this field.

### Theme 1: Community work strategies of social workers

Participants described community work strategies in their efforts to provide substance use services to young people. Subsequently, three sub-themes emerged: awareness campaigns, collaboration with local stakeholders and community dialogues.

#### Sub-theme 1.1: Awareness campaigns

Participants identified awareness campaigns as a strategic approach used to inform and educate community members about the potential dangers and consequences of substance use. These awareness campaigns often involve conducting workshops and providing information pamphlets to engage and raise community awareness effectively. In addition, social workers actively participate in awareness campaigns by organising and delivering informative presentations at local schools and clinics. These presentations aim to educate the community on important social issues, promote resources available for support and foster a deeper understanding of the challenges related to substance use. The following are selected examples of narratives from participants:

‘We do prevention or awareness campaigns; we invite them so that we can empower them by educating them about substance use.’ (Social worker, Participant 5, Female)

‘We regularly hold awareness campaigns to educate communities about the impact of substance use.’ (Social worker, Participant 7, Female)

In line with the research findings, Shadung (2024) highlighted the significant role of awareness campaigns in empowering social workers to disseminate vital information and knowledge about substance use within communities. These campaigns serve as crucial platforms that increase the visibility and accessibility of social work services, ensuring that community members are well-informed and engaged. By fostering a greater understanding of substance use issues, these initiatives inform the public and strengthen the connection between social workers and the communities they serve. In addition, Mathibela (2024) emphasised the need for social workers to implement awareness campaigns aimed at addressing the challenges faced by parents of young people recovering from substance use. It is crucial to highlight that, without greater community involvement, there may be lasting impacts on the community.

#### Sub-theme 1.2: Collaboration with local stakeholders

Participants actively collaborated with various local stakeholders as part of a community work strategy to tackle substance use. This collaboration involved working closely with organisations, government agencies and community leaders to develop and implement effective interventions that address substance use challenges within the community. The following are selected examples of narratives from participants:

‘As part of the community strategy to combat substance use locally, we formed a forum called the Local Drug Action Committee. This forum included other government Departments, the South African Police Service, community leaders, faith-based organisations, and non-governmental organisations.’ (Social worker, Participant 3, Female)

‘I was involved with the local drug action committee in our area. We met monthly with other stakeholders to plan activities and other ways of combating substance use in the community. This responsibility and experience have contributed a lot to my professional growth and my understanding of deeper problems of substance use within our community.’ (Social worker, Participant 8, Female)

The National Drug Master Plan (2019–2024) emphasises the vital significance of collaborative efforts among various stakeholders as a strategic approach to effectively combat the pervasive substance use issue across South Africa (DSD 2020). This collaboration aims to unite government agencies, non-governmental organisations, community groups and other relevant parties to create a comprehensive and coordinated response to the challenges posed by substance use within the country. By leveraging these stakeholders’ diverse expertise and resources, the plan seeks to implement innovative solutions and foster a supportive environment for individuals affected by substance use. Madisha (2019) underscored the importance of collaboration among multidisciplinary teams and various stakeholders in effectively providing social welfare services to young people using substances. This collaborative approach is essential for addressing the complexities of this issue.

#### Sub-theme 1.3: Community dialogues

Participants reported that as part of the community work strategy, community dialogues are held with the locals to get their views and solutions about their situation. These gatherings provide an open forum where individuals from the community come together to share their experiences, concerns and aspirations regarding substance use in the community. During these dialogues, participants are encouraged to voice their thoughts and discuss their daily challenges. The dialogues are seen as a crucial step towards building stronger community ties and empowering residents to take part in solving their community problems. The following are selected examples of narratives from participants:

‘We normally have community dialogues facilitated by the Soul City organisation.’ (Social worker, Participant 8, Female)

‘Discussions with individual members in the community are held to ascertain their perspectives on the current state of affairs.’ (Social worker, Participant 11, Male)

In line with the findings mentioned above, McEwan-Short (2023) highlighted that community spirit thrives where the voice and agency of individuals come to play in articulating community interests through structured dialogues to ensure social transformation and change. This engagement is vital for articulating the community’s diverse interests through well-organised dialogues. These structured discussions serve as a platform for collaboration and understanding, ultimately driving social transformation and facilitating meaningful change within the community.

### Theme 2: Support needs of social workers

Participants voiced their support needs in providing substance use services to young people. Six sub-themes were generated under the theme: the need for localised and easily accessible inpatient treatment centres, the need to reassess the current inpatient treatment programme, the need for additional social workers, the need for vehicles, the need for office spaces and the need for relevant training.

#### Sub-theme 2.1: Need for localised and readily accessible inpatient treatment centres

Participants underscored the critical need to establish localised inpatient treatment centres that are conveniently accessible to the communities they serve. They expressed that these centres should be strategically positioned within communities to minimise travel time and barriers to access. The availability of such facilities within the community is essential for timely and effective treatment services, which can significantly enhance the recovery process for needy individuals. In addition, these localised inpatient treatment centres would provide accessible services and support, ensuring that individuals can receive treatment without facing substantial geographical or logistical barriers:

‘We need to explore having inpatient treatment or rehabilitation centres locally. I found it as a matter of urgency. Ideally, we need to have a situation where we could refer substance users for rehabilitation immediately and possibly if they could walk or at least take a local taxi.’ (Social worker, Participant 1, Female)

‘All inpatient treatment centres are very far. We travel as far as Boksburg, Krugersdorp, Cullinan and Randfontein to get an in-patient treatment centre.’ (Social worker, Participant 9, Female)

In their study, Segal, Gerdes and Steiner (2016) confirmed that there are over 11,000 specialised drug treatment centres in the United States. However, there is still a significant shortage of these facilities. According to the authors, individuals seeking treatment for substance abuse may face waiting periods of up to six months in various metropolitan areas. While it might be unfair to compare a developing country to a developed one, this information highlights the magnitude of the shortage of treatment centres. Madisha (2019) pointed out that the scarcity of localised inpatient treatment centres in South Africa presents a significant challenge for social workers, as these facilities are frequently far from their clients. Consequently, social workers must travel considerable distances to obtain the necessary services for those they support.

#### Sub-theme 2.2: Need to reassess the inpatient treatment programme

Participants expressed the need to reassess the inpatient treatment programme to evaluate its effectiveness and suitability. This need arises from the observation that most individuals undergoing inpatient treatment for substance use do not complete the programme, which undermines its intended impact. Subsequently, this reassessment could lead to targeted improvements that would enhance engagement and support and ultimately help more individuals complete the treatment, thereby increasing the programme’s overall impact on recovery. The following are selected examples of narratives from participants:

‘First, we must assess and check the inpatient treatment program the users attend. It is important to check whether the program suits the users and establish why most prematurely terminate the treatment program.’ (Social worker, Participant 10, Female)

‘The success rate of the service user’s treatment program is very poor. For instance, out of ten users sent to an inpatient rehabilitation centre, one or two will complete the program and return home clean.’ (Social worker, Participant 7, Female)

In alignment with these findings, Mahlangu (2016) proposed a thorough reassessment of the duration of the inpatient treatment programme, recommending an extension from six weeks to six months. This adjustment aims to enhance the recovery process for substance users. Furthermore, the emphasis was placed on establishing employment opportunities for individuals who have completed the treatment programme, thereby supporting their reintegration into society and promoting sustainable post-treatment outcomes. Mathibela and Skhosana (2019) underscored the critical need for a greater number of treatment centres dedicated to addressing the challenges faced by young people dealing with substance use. They also advocated for the extension of treatment durations, arguing that longer therapeutic interventions would better support the recovery and rehabilitation of these young individuals, ultimately leading to more effective outcomes in their journey towards a healthier lifestyle.

#### Sub-theme 2.3: Need for more social workers

Participants called for more social workers to better address clients’ needs. They emphasised that a larger workforce would allow for more timely and practical support, ensuring that individuals facing various challenges receive the assistance and resources necessary to improve their circumstances. As a result, more social workers could enhance service delivery and promote positive outcomes for those in need. The following are selected examples of narratives from participants:

‘There are many clients who require assistance from a few social workers and, therefore, need more social workers.’ (Social worker, Participant 2, Female)

‘We need more social workers as we are currently not coping with the high number of clients requiring our help.’ (Social worker, Participant 8, Female)

In line with the findings, the study of Waini (2015) revealed that several social service organisations encounter difficulties recruiting additional social workers because of insufficient government funding. This situation underscores the need for government intervention to support these organisations financially. Accordingly, Mathibela (2024) highlighted that social service organisations are experiencing a significant shortage of social workers, which hampers their ability to respond effectively to the complex challenges faced by clients struggling with substance use issues. This understaffing creates barriers to providing timely and comprehensive support, ultimately impacting the overall effectiveness of interventions designed to help individuals overcome addiction and related challenges. The study highlights the urgent need for increased investment in workforce development within these organisations to enhance their capacity to serve this vulnerable population.

#### Sub-theme 2.4: Need for vehicles

Participants expressed a need for support, specifically in the form of vehicles. They highlighted that having access to vehicles would significantly enhance their ability to execute their responsibilities effectively. With vehicles at their disposal, they could travel to various locations, attend necessary meetings and complete their tasks promptly, ultimately improving their overall productivity and efficiency. Access to vehicles would facilitate their day-to-day operations and enable them to respond more swiftly to any challenges that may arise in their roles. The following are selected examples of narratives from participants:

‘The DSD in Gauteng needs to look at the possibility of getting subsidised cars for social workers to enable us to work effectively and reach out and attend to the service users and their families at their convenience. Having subsidised cars will mean there will be no need to book a car, but use the one in one’s possession at any given time to execute the professional work.’ (Social worker, Participant 7, Female)

‘We need resources such as cars to reach out to clients. Imagine a client crisis in the township we are servicing, but we cannot respond immediately due to a lack of resources. Most of the time, I use my car to service clients.’ (Social worker, Participant 10, Female)

Drawing upon the findings, Madisha (2019) emphasised the importance of enhancing the resources available to social workers by urging the DSD to provide additional vehicles. This increase in transportation would enable social workers to offer a broader range of comprehensive services, ensuring they can reach clients effectively and address their needs more efficiently. Accordingly, the study by Shadung (2024) highlighted an urgent need for vehicles to assist social workers in providing effective substance use services to clients. Consequently, vehicles would ensure social workers reach clients in need and deliver services more efficiently, ultimately improving substance use treatment and support outcomes.

#### Sub-theme 2.5: Need for offices

Participants emphasised the need for adequate office space. An office is essential for social workers because they work with vulnerable clients who share complex stories requiring confidentiality. Without appropriate office space, social workers may struggle to protect the sensitive information shared by their clients, potentially jeopardising the integrity of the client–worker relationship. A confidential office space allows social workers to focus intently on identifying each client’s unique challenges and providing tailored support. Thus, a need for such a space is paramount to the outcome of the social work intervention. The following are selected examples of narratives from participants:

‘Our office is crowded, and providing proper therapy under the setup is impossible. We need adequate office space to provide professional intervention to our vulnerable clients.’ (Social worker, Participant 3, Female)

‘I would appreciate it if the Department allocated each social worker an office space.’ (Social worker, Participant 4, Female)

The study by Sekgobela (2021) identified the need for office space as the primary requirement in the hierarchy of needs for social workers. This finding highlights the essential role that a suitable working environment plays in enabling social workers to perform their duties and support their clients effectively. In line with the findings, the study by Ntshongwana and Tanga (2022) revealed that it is common for social workers to share an office, as they often attend to clients in the presence of their colleagues. Additionally, during the summer, social workers bring their fans to the office, and in the winter, they bring their heaters because the department cannot provide these amenities. This situation highlights the need for the department to provide proper offices rather than compelling social workers to depend on personal solutions to work effectively and comfortably.

#### Sub-theme 2.6: Need for relevant training

Participants expressed the need to receive relevant training to acquire the skills and knowledge needed to effectively tackle the challenges and complexities that arise in the constantly evolving field of substance use. They believe that such training will help them stay informed. The following are selected examples of narratives from participants:

‘I think we can look at how social workers dealing with substance dependence can empower themselves in the form of training or workshops to help them counter substance dependence.’ (Social worker, Participant 5, Female)

‘We need the employer to assist us with resources and support, and identify relevant training. We usually attend substance abuse training, but only to learn that it is not relevant to what we are doing daily with our clients.’ (Social worker, Participant 7, Female)

The above responses correlate with the findings that there is a dire need for intensive training and resources to support social workers in executing their tasks effectively in the field of substance (Madisha & Skhosana 2022). Accordingly, the study of Mathibela (2024) revealed a need for comprehensive training interventions that equip social workers with the skills and knowledge needed to provide substance use services to clients effectively. These professionals play a crucial role in addressing substance use issues, and the study emphasised that enhancing their skills and knowledge is essential for them to carry out their responsibilities effectively in this challenging field.

## Discussion

The study aimed to explore the community strategies of social workers providing substance use services to young people. Furthermore, it explored the support needs of social workers providing substance use services to young people. The data collected reveal that social workers strategically implement awareness campaigns as a vital component of their community efforts to provide substance use services effectively. Through these campaigns, social workers strive to educate community members about the risks and detrimental effects linked to substance use. In line with the findings, Motsoeneng (2018) emphasises that social workers should utilise awareness campaigns to promote understanding of social policies. These policies are designed to safeguard the community from the effects of substance use. The study revealed that social workers are forming collaborative partnerships with various stakeholders within the community, such as local organisations, healthcare providers and law enforcement. These joint efforts aim to create a comprehensive approach to effectively combat substance use and its associated challenges within the community. By working together, they seek to leverage their resources and expertise to implement strategies that foster prevention, support and recovery for individuals affected by substance use. Related to the study, Bambeni and Engelbrecht (2024) emphasise the DSD’s importance in actively strengthening its partnerships with local authorities and various government institutions at the community level. The department can significantly enhance social development efforts by fostering these collaborative relationships, creating a more integrated approach to addressing communities’ needs and challenges. This strategic alignment has the potential to drive meaningful progress and foster a supportive environment for social growth and development. Accordingly, the study demonstrated that social workers utilise community dialogues as a fundamental aspect of their community work strategy. Social workers can enhance community cohesion by fostering open discussions among community members.

In addition, the establishment of localised and easily accessible inpatient treatment centres is of paramount importance. These centres serve as critical resources for social workers, enabling them to provide comprehensive substance use services tailored to the unique needs of young people within their community. By situating these facilities within the community, social workers can facilitate greater access to treatment, helping to reduce barriers that often hinder young people’s pursuit of necessary treatment. Research conducted by Masombuka (2021) and Nkangane (2022) highlights a significant disparity in South Africa regarding the accessibility of inpatient treatment centres, often far from certain townships. In contrast, taverns tend to be close to township schools, increasing the availability of substances for young people. This closeness not only facilitates access to these substances but also presents a substantial challenge to the well-being of learners within these communities.

The study underscored the critical need for a larger workforce of social workers, highlighting that increasing their numbers is essential for enhancing the effectiveness of substance use services. As a result, strengthening the workforce of social workers is essential for fostering a more comprehensive approach to addressing substance use issues. Putting it differently, the study of Sekgobela (2021) revealed that more social workers are needed to alleviate the daily challenges they experience. Accordingly, a call was made for resources in the form of vehicles and offices to assist social workers in their efforts to provide effective substance use services. Vehicles are crucial for transporting social workers to various locations, allowing them to reach clients who may need substance use services in remote areas. A private office space will also provide social workers with a professional environment to meet with clients and adequately conduct assessments without interruptions. By securing these resources, the authors aim to enhance the effectiveness and accessibility of substance use services, ultimately improving outcomes for individuals seeking assistance. Consistent with the findings, the study by Shadung et al. (2024) confirmed that social workers employed at the DSD encounter challenges related to inadequate resources, particularly regarding vehicles and office facilities.

Moreover, the study highlighted the importance of providing relevant training to social workers to enhance their ability to offer comprehensive substance use services. The relevant training would enhance their professional capabilities and ensure they remain informed about the latest research, best practices and innovative approaches in substance use treatment. In line with the findings, Madisha and Skhosana (2022) suggested that substance use is a growing and evolving social problem; therefore, social workers should also be continuously trained to expand their capacities and stay abreast by developing the necessary intervention skills. Equally, the study of Mathibela (2024) showed that social workers in the substance use field often lack the necessary training. This suggests that while there are high expectations for social workers, many of them are not adequately prepared to handle substance use cases. The gap between the expectations set for social workers and the training they receive underscores the need for enhanced training programmes and resources aimed at equipping these practitioners with the tools they need to confront the multifaceted challenges associated with substance use effectively.

### Limitations of the study

The study focused exclusively on social workers employed at the 10 service points of the DSD within the CTMM. This specific selection of participants allows for a detailed exploration of their experiences and practices; however, it also limits the applicability of the findings to a broader context. While the insights gained cannot be generalised to all social workers, they may offer meaningful implications for professionals in similar environments, such as those working in other provinces.

## Conclusion and recommendations

It was evident from the study that social workers implement community work strategies in providing substance use services to young people. Thus, the study concludes that awareness campaigns, collaboration with local stakeholders and community dialogues are pillars of social workers’ community work strategies in providing substance use services to young people. The recommendation for social workers is to expand community work strategies by incorporating peaceful marches and more community dialogues into their outreach efforts, fostering awareness and promoting solidarity within the community. In addition, the study highlighted the support needs of social workers in providing substance use services to young people. As a result, the study concludes that employing more social workers dedicated to substance use services will enhance the effectiveness of treatment outcomes. In this regard, a recommendation is put forth for the employer organisation to ensure that a sufficient number of social workers are employed to provide comprehensive substance use services to young people.

The study concludes that social workers require accessible inpatient treatment centres within local communities. These facilities are crucial for the effective and timely delivery of substance use services to young people. The study recommends establishing dedicated inpatient treatment centres in every community. The study concluded that inpatient treatment programmes should undergo a reassessment to ensure that they effectively address the needs of young people struggling with substance use. Thus, the recommendation is put forth for the adoption of evidence-based practices and a personalised treatment approach to assist young people battling with substance use.

It is also concluded that adequate, accessible vehicles and private office space designated for each social worker are important tools of the trade that social workers require to provide professional and effective substance use services. It is also recommended that the employer organisation provide accessible vehicles and private office space to enable social workers to provide substance use services professionally and comprehensively to young people. Furthermore, to effectively support young people dealing with substance use issues, social workers must participate in regular refresher training. This ongoing education is essential for deepening their knowledge and understanding of the complexities surrounding substance use, including emerging trends, effective intervention strategies and the social implications of substance use. Such training keeps their skills sharp and empowers them to serve their clients better and adapt to the evolving landscape of substance use intervention.
